# Recovery of Magnetic Catalysts: Advanced Design for Process Intensification

**DOI:** 10.1021/acs.iecr.1c03474

**Published:** 2021-09-22

**Authors:** Cristina González-Fernández, Jenifer Gómez-Pastora, Eugenio Bringas, Maciej Zborowski, Jeffrey J. Chalmers, Inmaculada Ortiz

**Affiliations:** † Department of Chemical and Biomolecular Engineering, ETSIIT, University of Cantabria, Avda. Los Castros s/n, 39005 Santander, Spain; ‡ William G. Lowrie Department of Chemical and Biomolecular Engineering, The Ohio State University, 151 W. Woodruff Avenue, Columbus, Ohio 43210, United States; § Department of Biomedical Engineering Cleveland Clinic 9500 Euclid Avenue, Cleveland, Ohio 44195, United States

## Abstract

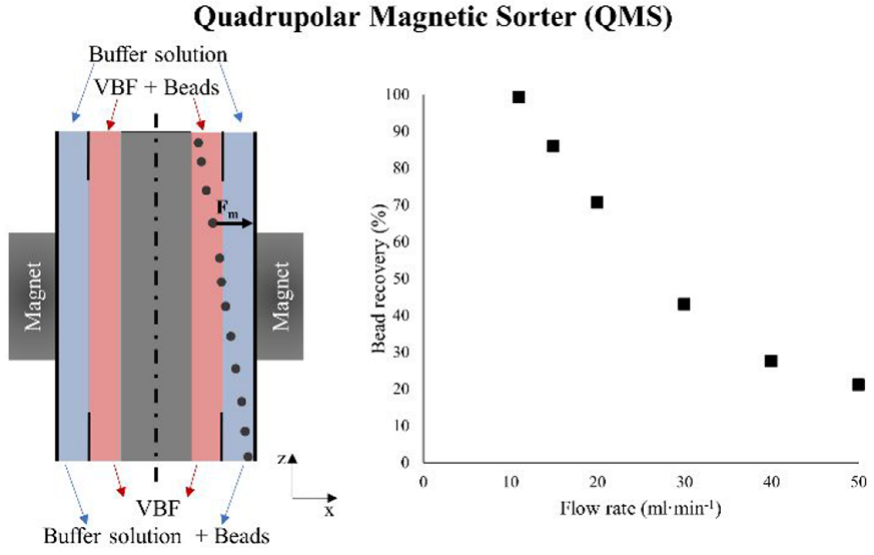

The design of microdevices in which components with magnetic character must be separated and recovered from reactive media benefits from the advantages of microfluidics and meets the criteria for process intensification; however, there are open questions, such as the design of the most appropriate magnet arrangement, that need further research in order to increase the magnetic gradient exerted on the particles. Herein, we focus on the continuous recovery of magnetic microparticles, that can be used as support to facilitate the recovery of biocatalysts (magnetic microcatalysts, MMCs) from biological fluids. We analyze and compare the performance of two typical magnetophoretic microdevices for addressing bead recovery: (i) annular channels with a quadrupole orientation of the permanent magnets (quadrupole magnetic sorter, QMS) and (ii) the standard design, which consists of rectangular channels with a single permanent magnet to generate the magnetic field. To this end, an experimentally validated computational fluid dynamics (CFD) numerical model has been employed. Our results reveal that for devices with the same width and length, the micro-QMS, in comparison to a rectangular channel, could accomplish the complete particle retrieval while (i) processing more than 4 times higher fluid velocities, treating more than 360 times higher flow rates or (ii) working with smaller particles, thus reducing by 55% the particle mass. Additionally, the parallel performance of ≈300 micro-QMSs fulfills the processing of flow rates as high as 200 L·h^–1^ while entirely capturing the magnetic beads. Thereby, this work shows the potential of the QMS advanced design in the intensification of the recovery of catalysts supports of magnetic character.

## 1 Introduction

Functionalized magnetic micrometer-sized or nanosized particles, also referred to as beads, offer promising possibilities in catalytic reactive systems that involve biomolecules; thus, magnetic microparticles have received special attention to be used as biocatalyst and enzyme supports (magnetic microcatalysts, MMCs).^1−3^ The applicability of MMCs in catalytic reactions stems from their interesting properties. Specifically, they exhibit high surface-to-volume ratios and loading capacities, as well as chemical stability and biocompatibility after being submitted to several surface treatments. Such enhanced features lead to the reduction of both the costs and the generated contamination, and to the increase of the process selectivity when using MMCs in comparison to conventional materials. Additionally, the recovery of the MMCs from the reaction media can be easily accomplished through the application of magnetic fields, since the superparamagnetic behavior of the magnetic beads enables their manipulation with simple permanent magnets.^1,4−7^

Intensifying catalytic reacting processes that use magnetic beads as support for biocatalysts or enzymes can be accomplished by performing such processes in microfluidic platforms, thus taking advantage of the unique characteristics of microfluidics, which meets process intensification through miniaturization.^3,8−13^ Thereby, scaling down the dimensions of fluidic channels to the microscale increases the surface-to-volume ratio of the fluids, which in turn leads to an enhanced mass transfer rate. Furthermore, the laminar flow developed in microstructures allows for a precise control of the fluid flow.^5,14−16^ On the other hand, the small dimensions of microchannels also involve low-sample consumptions, thus reducing the risk of managing hazardous materials and the use of expensive reagents; accordingly, the decrease of the amount of waste that is generated and a considerable cost saving are accomplished. The use of small volumes also facilitates the control of process parameters, such as temperature, pressure, and residence time, etc.^8,14,17,18^

Different types of microreactors have been used for performing reactions that are catalyzed by enzymes supported in magnetic beads. From all of them, microreactors with an oscillating magnetic field have received special attention due to the enhanced mixing patterns that can be created.^3,19,20^ By allowing the movement of magnetic beads in the reactor through the application of an oscillating magnetic field, particles can cover the entire channel cross-section, thus, increasing the availability of the enzyme to the substrate. In these systems, the dual role of the magnetic beads is noticeable, since they are not only used as enzyme carriers but also for enhancing mixing.^19,21−23^ For example, Šalić et al.^22,24^ performed the oxidation of nicotinamide adenine dinucleotide hydrate (NADH), catalyzed by the alcohol dehydrogenase enzyme supported on magnetic nanoparticles, in a microreactor equipped with an electromagnet that generated the oscillating magnetic field. Once the reaction completes, the recovery of the particles is required in order to obtain an enzyme-MMCs free stream with the target product, as well as to recycle MMCs for further uses. Taking advantage of the magnetic nature of the MMCs, particle recovery can be easily fulfilled by magnetic means. Nevertheless, the batch recovery of particles in the reaction chamber poses several drawbacks, such as the particle aggregation, which hampers their reuse, the restriction of the fluid flow, or the entrapment of nontarget compounds. These shortcomings ultimately result in the decrease of the particle recovery efficiency. Conversely, surmounting the limitations of batch systems by addressing the MMCs isolation in continuous systems is encouraged in order to increase the efficiency of particle isolation. To this end, taking advantage of the outstanding features of microfluidics, a second stage after the catalytic microreactor can be included in order to perform the continuous isolation of MMCs, since microfluidics enables the integration of several steps within the same device.^4,5^

The design of continuous-flow microfluidic-magnetophoretic devices to perform the recovery of the magnetic materials has been addressed both experimentally and theoretically.^5,25−27^ In this regard, the effect of numerous variables, such as fluid flow rates, fluid rheological properties, particles size, magnet dimensions and positions, etc. on the performance of magnetophoretic-microrecovery systems has been elucidated.^26,28−31^ On the other hand, there is huge interest in optimizing the performance of such systems (i.e., obtaining complete recoveries when processing relatively high flow rates) so they could be effectively employed; to this end, the impact of the channel geometry on the particle recovery has also been investigated.^5,32,33^ In our previous work,^5^ we demonstrated the effect of the cross-section shape and thickness, which depend on the method used for the channel fabrication, as well as of the channel length and volume on the performance of these systems. Besides, we identified from the vast number of studied geometries the one that exhibits the best performance; thereby, we found that long microchannels with rectangular cross sections enable the processing of flow rates up to 2 orders of magnitude higher than other geometrical configurations while entirely capturing the beads. However, channel lengthening is insufficient for considerably increasing the treated flow rate while providing complete particle capture. In this regard, further optimization of the performance of magnetophoretic-microrecovery systems relies on increasing the driving force for the isolation of the particles, that is, the magnetic force exerted on the beads. Generating regions with high magnetic field gradients has become a potential strategy for enhancing the efficiency of these systems. To this end, the use of several permanent magnets arranged in a quadrupolar orientation, which gives rise to a quadrupole magnetic sorter (QMS), represents an outstanding alternative since field gradients higher than those typically reached by a single permanent magnet could be obtained with QMSs.^1,34−37^ Thereby, several QMSs have been designed and tested over the years for carrying out the magnetic isolation of both not labeled and labeled cells with magnetic beads.^35−45^

In this work, we further optimize continuous-flow magnetophoretic-microfluidic systems for magnetic microparticles recovery. Two different designs of typical recovery devices are tested: (i) a device with a single permanent magnet and, (ii) a device with a quadrupolar magnet configuration. The latter, which will be referred to as micro-QMS, could be integrated after the catalytic microreactor in a microfluidic platform for efficiently recovering and recycling the MMCs. Particularly, we enhance the isolation of micrometer-sized magnetic particles from a viscous biofluid that flows along an annular flow channel and their recovery into an aqueous buffer solution. We demonstrate and quantify the improvement of the system efficiency when a quadrupole magnetic field is applied in comparison to the use of a single permanent magnet for channels with the same geometrical features. We also design a system based on several micro-QMSs in parallel in order to increase the flow rate that can be processed while completely recovering the beads so as to these systems could be exploited to fulfill industrial needs. Finally, since the reduction of particle size positively influences the adsorption of target molecules onto the beads, we determine the reduction in the size of the particles that could be affordable to ensure an acceptable performance of the previously designed micro-QMS. Collectively, this study demonstrates the improved performance of QMSs, which substantiates their suitability to be used after catalytic reactions in microreactors, and also provides the basis for further optimization of the QMS systems.

## 2 Computational Model

### 2.1 Modeling Approach

In this work, we employed our previously derived computational model.^5,26^ The model predicts the transport of magnetic beads through an Eulerian-Lagrangian approach. The particle trajectory is computed according to the following equations by taking into account the dominant magnetic (**F**_m_) and hydrodynamic (**F**_hd_) forces acting on the beads:
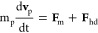
1
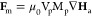
2
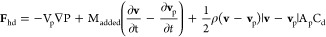
3where m_p_ and d**v**_**p**_/dt are the mass and acceleration of the magnetic beads, μ_0_ is the permeability of the free space (4π × 10^–7^ H·m^–1^), V_p_ and M_p_ are the bead volume and magnetization and **H**_a_ is the magnetic field that is applied at the particle center. P, **v**, and M_added_ represent the pressure, the fluid velocity, and the added mass, which is equal to 0.5ρV_p_, with ρ representing the fluid density. Finally, A_P_ is the particle cross sectional area and C_D_ stands for the drag coefficient for a steady-state flow around a sphere.^46,47^

Herein, we simplify the investigation of MMCs magnetophoresis by considering that the particles comprise both the catalyst and the magnetic support, thus, the presence of catalyst is contemplated in the bead size; future studies will take into account the morphological and structural variations of catalyst–supported beads. Thereby, spherical, micrometer-sized beads with different diameters (4.9, 2.45, 2.22, and 2 μm) have been considered. Regardless of the particle size, beads are assumed to have the same density, which is equal to 2000 kg·m^–3^. The magnetization of the particles is estimated by using saturation magnetization and susceptibility values of M_s,p_ = 1.5 × 10^4^ A·m^–1^ and χ_p,e_ = 0.25, respectively, which fall within the range of commercially available beads.^26,46^ Regarding the magnetic properties of the surrounding fluids, they are considered nonmagnetic, since their susceptibilities are considerably lower than that of the particles. To evaluate **F**_hd_, both the velocity profile and the properties of the fluids are required. Our analysis involves two fluids: a viscous biofluid (VBF) and an aqueous buffer solution. We modeled the VBF as a Newtonian fluid with a viscosity equal to 3.5 cP in order to take into account the effect of fluid viscosity on particle magnetophoresis, and the aqueous buffer solution as water with a viscosity value of 1 cP. Regarding the fluid velocity field, it is estimated by solving the Navier–Stokes and continuity equations for incompressible flows, taking into account the impact of magnetic beads motion on the fluid flow via a two-way momentum exchange.^5,26^

Finally, our theoretical model was validated by simulating a QMS system reported in the literature,^37^ that has been experimentally employed for recovering a paramagnetic material (deoxygenated red blood cells, with a size similar to the magnetic beads employed in this work, and with a known magnetization and size/volume distribution, which have been widely reported in the literature).^47,48^ Our model results resulted to be in good agreement with the experimental data for several flow rate values, as can be observed in the Supporting Information (Figure S1).

### 2.2 QMS Description and Simulation Setup

**Figure 1:**
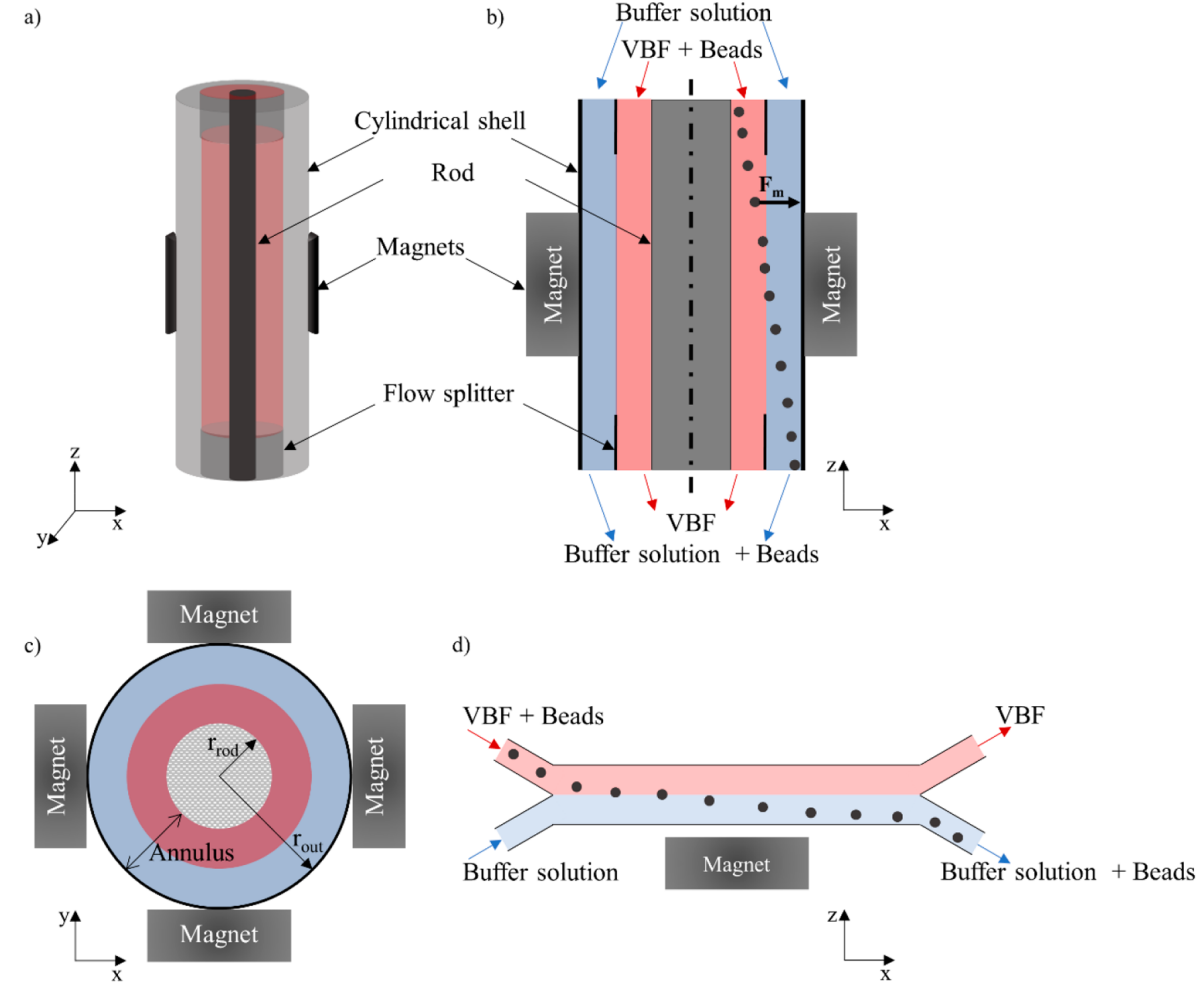
(a) Perspective view, (b) longitudinal section, and (c) cross-section, of the micro-QMS; (d) longitudinal section of the conventional microrecovery system.

The micro-QMS comprises essentially two components, namely, the flow channel and the magnets.^37,39^ The flow channel, where the recovery of the magnetic beads takes place, consists of an annular channel; specifically, it is composed of a cylindrical shell in the external side that is concentric with a cylindrical rod located at the center, as it is illustrated in Figure 1a. As it has been represented in Figure 1b, the flow channel has inlet and outlet manifolds that allow for the injection of the magnetic particles suspended in the VBF and the buffer solution at the system inlet, as well as the collection of the magnetic beads free VBF and the magnetic beads enriched buffer solution at the outlet.^37,39^ Once injected, the VBF and the buffer flow through the annular channel; particularly, the VBF flows through the half of the annular channel that is closest to the rod, whereas the buffer solution coflows through the other half of the annular channel (closest to the magnet poles). Magnetic beads, which are injected through the same inlet as the VBF, migrate radially between r_rod_ and r_out_ (see Figure 1b,c) and are collected in the coflowing buffer stream by applying a magnetic field.^37−39^ The dimensions of the annular channel under investigation are listed in Table 1. These geometrical features correlated well with those of other QMSs reported in the literature.^35−40^ It is worth mentioning that the dead volume of the flow channel (V_dead_) has been calculated using r_rod_. The r_rod_ value should be carefully selected in order to design systems with a V_dead_/V_total_ high enough to work at magnetic field values that maximize the particle magnetization (i.e., saturate the particles). Thus, the V_dead_/V_total_ value needs to be optimized to ensure both the beads saturation and the processing of relatively high flow rates (see Figure S2). Additionally, in order to effectively compare the performance of the micro-QMS and our previously designed Y–Y rectangular microrecovery system (Figure 1d) that uses a single permanent magnet to generate the magnetic field (hereafter, conventional microrecovery system), the channel length and annulus of the micro-QMS were chosen to be the same as in the conventional system (see ref (5)). It is worth mentioning that 10 mm long channels (QMS and conventional microrecovery system) have been considered herein since from the different geometries we tested in our previous study^5^ conventional systems with that length provided the best performance.

**Table 1:** Channel and Magnet Parameters of the Micro-QMS System

parameter	value
rod radius, r_rod_, (mm)	2.54
outer QMS radius, r_out_, (mm)	2.84
annulus (μm)	300
QMS length, L, (mm)	10
V_dead_/V_total_ (−)	0.8
magnet dimensions (length × height × width) (mm^3^)	10 × 5 × 3
maximum field, **B**_0_, (mT)	530.7
magnetic field gradient, ∇**B**, (T·m^–1^)	186
maximum energy product of permanent magnets, (B × H)_max_, (kJ·m^–3^)	350

On the other hand, the magnetic field that drives the particle recovery is provided by four permanent magnets arranged in quadrupolar orientation that surround the annular channel.^37,39^ Magnets with the same dimensions as the one we employed in our conventional microrecovery system (10 × 5 × 3 mm^3^), which is commercially available, have been considered. These magnets produce a quadrupole field with a maximum field (**B**_0_) at the pole tips (**B**_0_ = 530.7 mT) and a constant field gradient equal to 1.5 × 10^8^ A·m^–2^ (or 186 T·m^–1^) through the QMS axial section. Since the maximum field in the micro-QMS matches the magnetic field on the pole surface of the permanent magnet considered in the conventional system, comparing the performance of both systems can be suitably addressed. Additionally, the maximum energy product of each magnet (see Table 1) has been provided by the commercial vendor.

Regarding the simulation setup, the force balance acting on each particle was solved using a 3D analysis, since the magnetic force was computed in *x*, *y*, and *z* directions. However, the governing equations that describe the flow were solved in 2D (only the radial and axial components were computed). A mesh independence study was performed in order to optimize the number of cells considered in the simulations; hence, a trade-off between the accuracy of the results and the computational cost of the simulations was achieved by using a mesh that comprises approximately 1 000 000 cells.

For investigating the particle magnetophoresis in the micro-QMS system under different flow conditions, the VBF was injected at velocities varying between 1.6 and 70.3 cm·s^–1^, which results in a flow rate range of 2.5–104 mL·min^–1^. These velocities for each flow rate were used as initial conditions. With respect to the boundary conditions, we applied a no slip condition (zero velocity) along the walls of the micro-QMS, and at the outlet, the outflow boundary was used. Depending on the inlet velocity value of the VBF, a particle flow rate between 2000 and 8000 particles·s^–1^ was considered, which corresponds to a concentration value between 0.6 and 1.48 mg·L^–1^. Beads were randomly introduced into the cross section of the VBF inlet, as depicted in Figure 1, with the same velocity as the VBF. A simulation time lower than 2.5 s was kept for all cases.

The commercial Computational Fluid Dynamics (CFD) software *FLOW-3D* (version 11.2, Flow Science, Inc.) was used to solve the computational model. Specifically, the *FLOW-3D* solver was linked to a customized FORTRAN subroutine compiled in *Visual Studio* 2013 (Microsoft), that allows for the calculation of the magnetic force and field distribution. All simulations were performed on a 48-core workstation with 128 GB of RAM.

### 2.3 Dimensionless Analysis

In this work, two dimensionless parameters were exploited in order to elucidate the effect of both the force balance acting on the beads and the channel geometry, and also to effectively compare the performance of the micro-QMS with the conventional microrecovery system. The parameter **J** balances the magnetic and drag forces that are exerted on the beads in the radial and axial directions, respectively. This parameter, which accounts for the magnetic and fluidic variables and parameters that affect the bead trajectory, can be written as follows:^49^
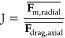
4To calculate the drag force, the viscosity and mean velocity of the VBF were considered, given that a successful bead recovery involves the transfer of the particles from the VBF, where they are originally injected, to the buffer stream.

The second parameter, θ, relates the residence time of the beads in the device (t_res_) and the time they need to migrate from the VBF to the buffer stream considering that they move purely in the magnetic field direction, that is, perpendicular to the fluid flow (t_m_). θ not only comprises the variables and parameters considered in **J**, but also includes the channel aspect ratio, that is, channel length (L)/annulus (or width for the conventional system, W,) ratio. Hence, this parameter can be described as:^5^
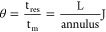
5On the other hand, the system performance is assessed by calculating the bead recovery that can be accomplished, and the exploitation of the magnetic energy for obtaining complete bead recoveries (φ). Specifically, bead recovery is defined as the percentage of particles that are collected in the buffer stream compared to the total number that are introduced in the device. Under the flow rate conditions employed in this work, permanent magnetic particle trapping at the channel walls is negligible: all the particles are collected in the buffer. In fact, and even at the lower flow rate values simulated, the particles that reach the channel wall next to the magnet pole roll and eventually exit the device within the buffer. Thus, the particle recovery can be calculated as follows:
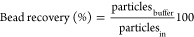
6Additionally, φ, in units of m·s^–1^·kJ^–1^, is a measure of the velocity at which fluids can be injected in the microchannel to achieve complete particle retrieval, per unit of energy generated by the magnet arrangement. Such energy is computed as the product of the maximum energy product of the magnet, (B × H)_max_, and the volume of the magnet arrangement, *V*_magnet_.
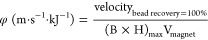
7

## 3 Results and Discussion

### 3.1 Influence of Magnetic Field Gradient

In this section, the performance of the micro-QMS under investigation, whose geometrical features have been included in Table 1, is characterized and compared to a device in which a single permanent magnet is used. As it has been previously mentioned, the arrangement of four permanent magnets in quadrupolar orientation leads to a considerable increase in the magnetic field gradient generated inside the QMS. Specifically, magnetic field gradients of about 1.5 × 10^8^ A·m^–2^ (or 186 T·m^–1^) are generated inside the micro-QMS, which result in magnetic forces acting on the beads of 0.17 nN. This magnetic force is one order to magnitude higher than the one generated in the conventional system operated with a single permanent magnet (around 0.04 nN). It is worth mentioning that although this difference in the magnetic force mainly stems from the higher magnetic field gradients in the micro-QMS, the distance between the magnet and the flow channel, which is higher for the conventional system (200 μm), should be also considered. Thereby, in the conventional system ensuring a channel-magnet distance is required since otherwise the magnetic field gradient would be generated only around the magnet corners (at the channel inlet and outlet) with zero magnetic force along the channel length; moreover, the limitations of the methods employed for fabricating these systems hamper the magnet to be located next to the channel wall.

**Figure 2:**
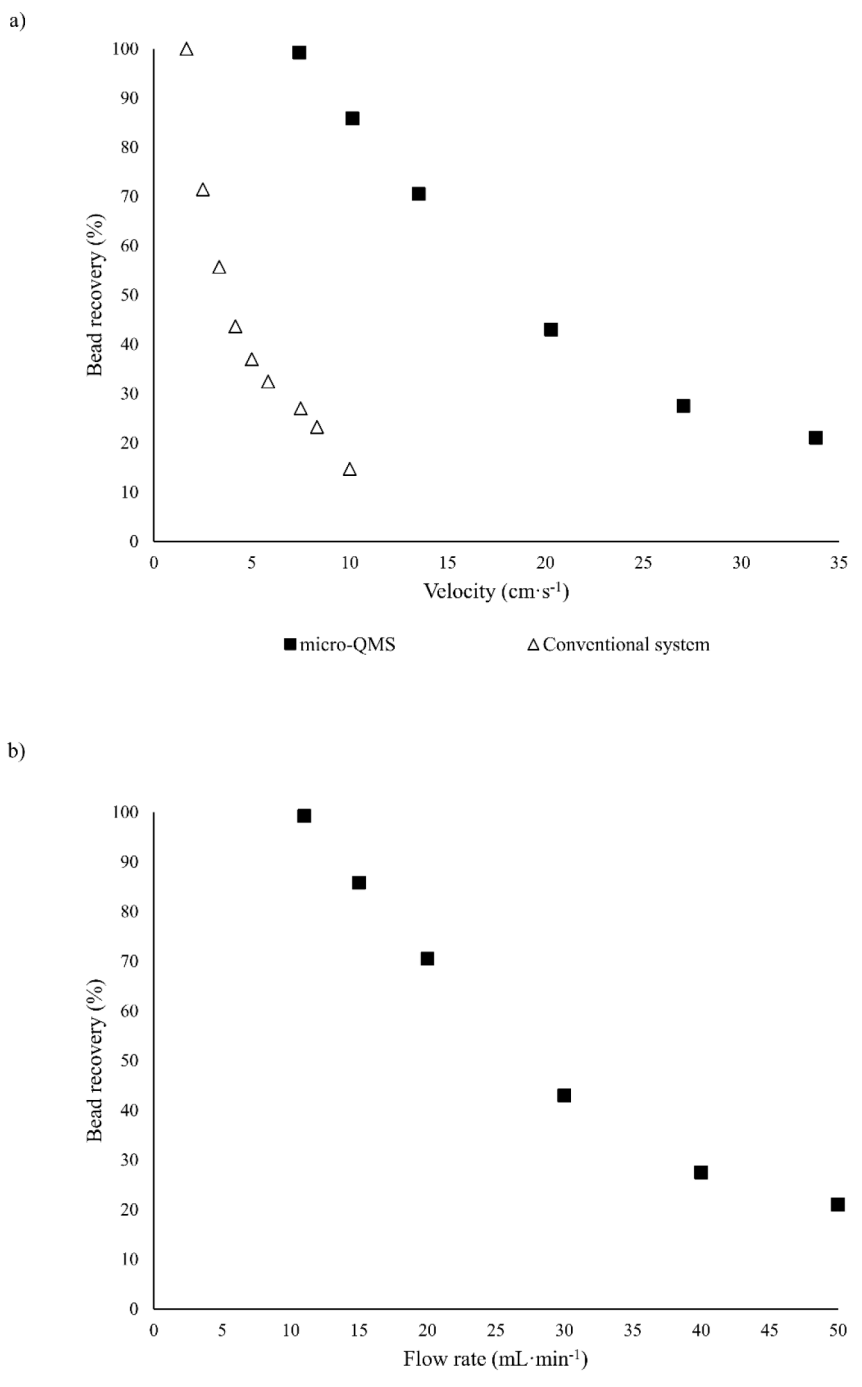
Dependence of particle recovery on VBF (a) velocity in the micro-QMS system and in the conventional system, and (b) flow rate in the micro-QMS (the flow rates that can be processed by the conventional system are 3 orders of magnitude lower).

As a result of this high magnetic force, the fluids can also be injected in the micro-QMS at higher velocities while ensuring complete particle capture. In Figure 2a, the percentage of particle capture as a function of the VBF inlet velocity is depicted. It can be seen that complete isolation of magnetic beads is fulfilled for fluid velocities of 7.44 cm·s^–1^. This velocity is approximately 4.5 times higher than the one that enables the complete particle capture in our conventional system that uses a single permanent magnet for generating the magnetic field. Therefore, for systems with the same cross-section area, the QMS allows the treatment of flow rates 4.5 times higher than conventional microseparators, hence, leading to a significant enhancement of the system efficiency. Figure 2b illustrates the bead recovery as a function of the processed VBF flow rate. As it can be easily noticed, complete recovery of magnetic beads in the micro-QMS is accomplished while treating VBF flow rates as high as 11 mL·min^–1^, which is 367 times higher than the one processed in our conventional microrecovery system (0.5 μL·s^–1^). Thus, the micro-QMS significantly enhances the VBF flow rate that can be treated while providing complete bead capture; this high flow rate stems from both the larger cross section area of such system and the bigger magnetic field gradient achieved when 4 magnets are employed, which allow the processing of higher velocities in comparison to the conventional system. Therefore, increasing the magnetic field gradient entails a noteworthy enhancement of the system performance in comparison to the lengthening of conventional microrecovery systems, which represents another strategy fulfilled in the literature for improving the efficiency of particle retrieval.^5^ Thereby, increasing the channel length of conventional systems from 2 mm to 10 mm involves only a 5-fold increase of the VBF flow rate that could be treated while entirely capturing the beads, which significantly contrasts with the ability of the QMS to treat 3 orders of magnitude higher flow rates in comparison to the conventional system. Thus, the micro-QMS exhibits an outstandingly improved efficiency, which substantiates its potential for being used for the recovery of magnetic beads when relatively high flow rates are required. These flow rates can be further increased by using higher magnetic field gradients, such as the ones considered in several QMSs published in the literature, namely, 300 T·m^–1^ or 1750 T·m^–1^.^34,37^ Assuming that these magnetic gradients can be ensured in the micro-QMS presented here (or one with a similar cross sectional area) by employing permanent magnets with a higher maximum magnetic field at their pole tips, and keeping the magnetic field value high enough to saturate the particles, complete bead capture can be fulfilled at flow rates of 18 mL·min^–1^ and 104 mL·min^–1^; these values correspond to flow rates of approximately 1.6 (∇**B** = 300 T·m^–1^) and 9.5 (∇**B** = 1750 T·m^–1^) times higher than the ones than can be processed when using the 186 T·m^–1^ gradient employed in this study. However, increasing the magnetic field gradient by reducing the QMS radius (instead of increasing the maximum magnetic field) could entail an undesirable decrease of the flow rate that can be processed for providing entire bead capture. Indeed, the decrease of the QMS cross sectional area dominates the effect of increasing the magnetic field gradient exerted on the particles (see Figure S3). Collectively, the arrangement of magnets with high magnetic fields in their pole tips entails the application of higher magnetic field gradients, thus, enhancing the efficiency of the micro-QMS.

### 3.2 Effect of Particle Size

**Figure 3:**
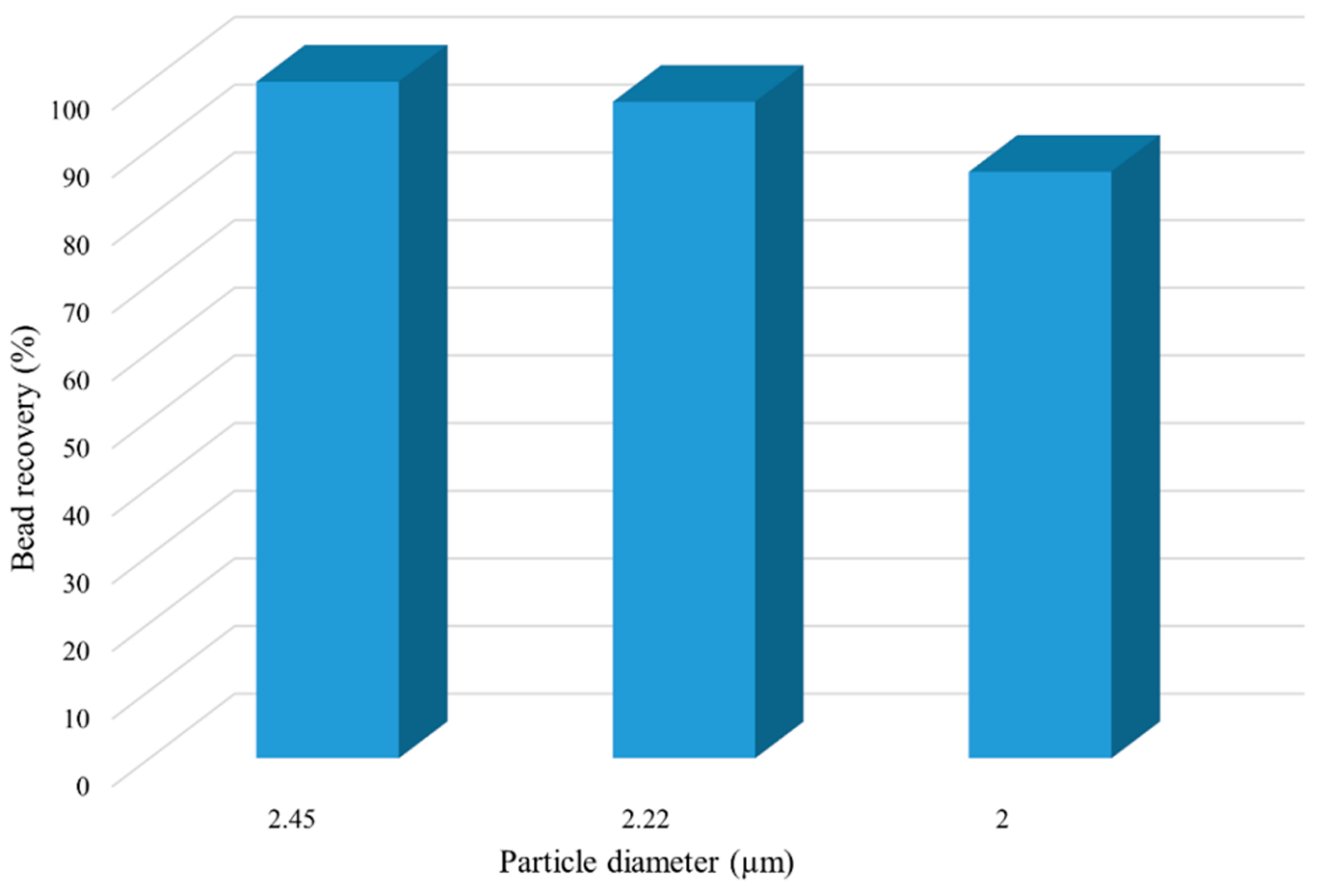
Effect of particle diameter on bead recovery in the micro-QMS.

Reducing the dose of magnetic beads that is required for supporting the desired amount of biocatalysts or enzymes in the reaction media seems promising in order to further fulfill the criteria of process intensification and to reduce the process costs. This fact could be addressed by decreasing the particle size, which leads to an increase of its specific surface area, and thus of the amount of biocatalyst or enzyme than can be adsorbed on the surface of the particles. Conversely, the magnetic bead recovery after the reaction process is favored by increasing the particle size, as it has been previously demonstrated.^26^ Therefore, and by taking advantage of the improved performance of the micro-QMS, we assess the reduction in particle size that can be accepted while achieving complete bead captures at relatively high fluid velocities in comparison to the use of particles with a diameter of 4.9 μm. To this end, we investigate the magnetophoresis of particles with different sizes when they are injected in the micro-QMS at the same velocity as the one that yields complete recoveries in the conventional system. As seen from Figure 3, the complete recovery is fulfilled in the QMS when 2.45 μm particles are used, whereas to achieve the same recovery with the conventional system 4.9 μm particles are required; thus, halving the particle size does not lead to worsen the micro-QMS performance. Further reduction of particle size (2 μm) results in an undesirable decrease of bead retrieval in the micro-QMS. Therefore, reducing bead diameter beyond 2.22 μm proves unaffordable, since particle recoveries lower than those obtained with the conventional system are accomplished. The specific surface area of 2.22 and 4.9 μm particles is 1.35 m^2^·g^–1^ and 0.61 m^2^·g^–1^, respectively. Hence, as it was previously mentioned, smaller particles enable the adsorption on their surface of higher amounts of the biocatalyst or enzyme, thus favoring the catalytic process. Additionally, the mass of particles required to achieve a surface area equal to 1 m^2^ is 0.74 g for the 2.22 μm and 1.63 g for the 4.9 μm particles. Therefore, the use of 2.22 μm beads entails a 55% reduction of the particle mass for achieving the same surface area as 4.9 μm beads. Since complete retrieval of 2.22 μm beads can be accomplished in the micro-QMS, the improved performance, meeting the criteria for process intensification of the QMS over the conventional system, is demonstrated.

### 3.3 Dimensionless micro-QMS Characterization

**Figure 4:**
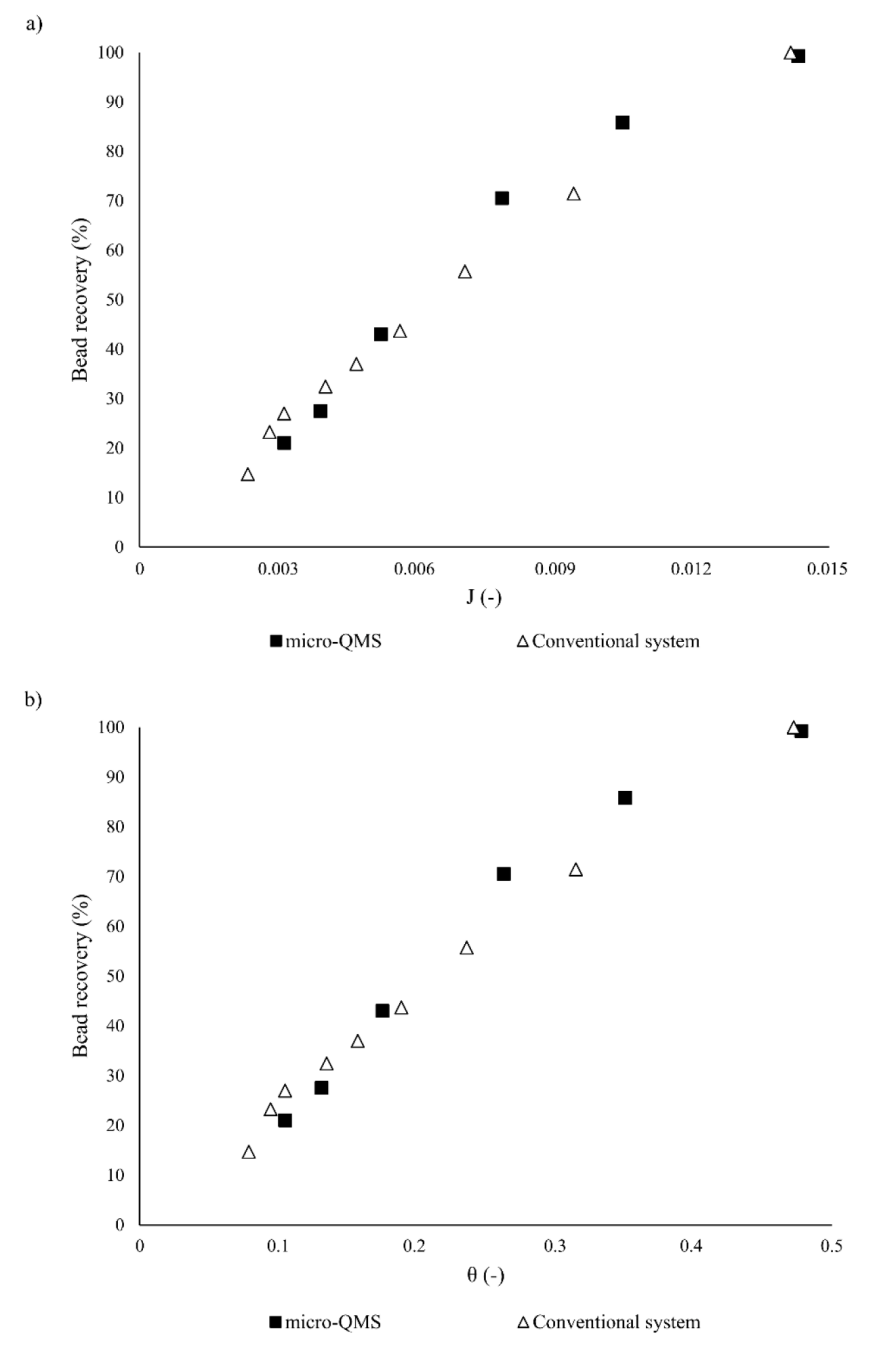
Magnetic bead retrieval as a function of the dimensionless parameters (a) J (balance of magnetic and drag forces), and (b) θ (coupling of magnetic and fluidic forces with the channel geometry).

In this subsection, the aforementioned parameters J, θ, and φ will be applied to characterize the performance of the micro-QMS. The parameter J allows determining the ratio of forces, namely magnetic and drag, that should act on the beads in order to successfully achieve the desired particle recovery. When studying the influence of the fluid velocity on the micro-QMS performance, the magnetic force is the same in all scenarios, since the magnetic field gradient and particle size are held constant for all the simulations. Conversely, the drag force changes with the velocity at which the VBF is injected in the micro-QMS. As it can be seen from Figure 4a, where the percentage of bead recovery has been represented as a function of J, the recovery of magnetic beads is favored by increasing J. This results from the decrease in the velocity, and thus the drag force, that is required for achieving complete particle capture. Regardless of the fluidic conditions, J-values lower than 1 are derived, which implies that higher drag forces than magnetic ones can be exerted on the beads while attaining entire recoveries. Particularly, the J-value that yields complete bead capture is 0.014, which is the same as the one for accomplishing the same recovery in the micro-QMS when different magnetic field gradients (300 T·m^–1^ or 1750 T·m^–1^) are used, as well as in the conventional microrecovery system. Since the J-parameter is directly proportional to the magnetic force and scales inversely to the drag force, these similar J-values regardless of the magnetic field gradient or the channel geometry system stem from the compensation of the magnetic and drag forces.

On the other hand, when investigating the effect of the particle size on the micro-QMS performance, the magnetic force changes with the size of the magnetic bead, although the same magnetic properties of the particles have been assumed for all the simulations. Moreover, particles with different diameters are subjected to different drag forces although the fluid velocity has been kept at the same value (1.67 cm·s^–1^). Thereby, reducing the particle size diameter leads to a decrease of the magnetic and drag forces exerted on these beads. It is worth mentioning that reducing the magnetic force negatively influences particle retrieval, whereas decreasing the drag force has a positive effect on it. According to eqs 1–3, particle size has a higher impact on the magnetic than on the drag force; hence, for the same fluidic conditions, lower J-values are expected when reducing the particle diameter. Thereby, the J parameter for 2.45, 2.22, and 2 μm beads when the above-mentioned fluid velocity is applied takes the values of J_2.45_ = 0.016, J_2.22_ = 0.013, and J_2_ = 0.011. Since it was previously demonstrated that reducing the particle diameter beyond 2.22 μm proves unaffordable due to the unacceptable recovery that could be accomplished, we conclude that bead size should be carefully chosen so that J-values around 0.013 could be obtained. Collectively, it can be rationalized that, regardless of the magnetic field gradient or the particle size, the improved performance of the micro-QMS over the conventional microrecovery system is maintained while ensuring J-values around 0.014.

Following the dimensionless analysis, the parameter θ was exploited to elucidate the simultaneous influence of the channel geometry and the magnetic and fluidic conditions inside the micro-QMS. The dependence of particle recovery with θ (Figure 4b) shows the same trend as with J; hence, particle recovery increases with θ. Additionally, θ values lower than 1 are obtained for all the fluidic conditions we have tested. Specifically, when θ is approximately 0.5, complete particle capture is fulfilled, regardless of the magnetic field gradient applied to the beads (186 T·m^–1^, 300 T·m^–1^, or 1750 T·m^–1^); according to the definition of the θ parameter (see eq 5), this θ-value denotes that magnetic beads can be entirely recovered by ensuring that the residence time of the particles in the device is one-half of the time they need to go from the VBF to the buffer stream considering that their movement is completely perpendicular to the flow direction. Given that the J parameter and the channel aspect ratio are the same for the micro-QMS and the conventional system, as it has been previously discussed, the same dependence of the percentage of bead recovery may be expected for these geometries.

Regarding the comparison of the θ parameter for beads with different sizes when an inlet fluid velocity of 1.67 cm·s^–1^ is used, we can conclude that the θ-value for obtaining complete recoveries of 2.45, 2.22, or 2 μm beads decreases with the particle diameter, θ_2.45_ = 0.53, θ_2.22_ = 0.44, and θ_2_ = 0.36. Since smaller particles than 2.22 μm could not be entirely retrieved in the QMS while using the above-mentioned inlet velocity, particle diameters that yield θ-values around 0.5 should be selected in order to ensure the outstanding performance of the micro-QMS in comparison to the conventional microrecovery system.

Finally, we compare the efficacy of the exploitation of the energy generated by the magnet arrangement for attaining complete recoveries in the micro-QMS and in the conventional system. To this end, the φ parameter is employed. As it was previously stated, the same magnets were used in both systems; thus, the maximum energy product and the volume of each magnet does not change for these systems (see Table 1). However, since the magnet arrangement in the QMS comprises four permanent magnets, the volume of the magnet arrangement in the QMS is 4 times higher than that in the conventional system, where solely a single magnet is used; therefore, the magnetic energy generated in the QMS is four times higher than in the conventional system. Because of this higher magnetic energy, fluids can be injected at considerably higher velocities (approximately 4 times higher) in the QMS than in the conventional system while fulfilling the entire particle retrieval, as it was previously discussed in section 3.1. However, if the fluid velocity for fulfilling complete recoveries per unit of magnetic energy (φ parameter) is contrasted for the micro-QMS (φ_QMS_ = 3.54 × 10^2^ m·s^–1^·kJ^–1^) and the conventional system (φ_conventional_ = 3.17 × 10^2^ m·s^–1^·kJ^–1^), it can be noted that both systems exhibit a similar efficiency in the exploitation of the magnetic energy, since the high fluid velocity in the QMS is compensated with its high magnetic energy, and the low fluid velocity in the conventional system counterbalances the low magnetic energy in this system. Collectively, when the performance of the micro-QMS and the conventional system per unit of magnetic energy that they exploit are assessed, it can be concluded that both systems enable the injection of the fluids at the same velocity for fulfilling complete bead retrieval; however, the flow rate that can be processed in the micro-QMS while attaining such recovery is outstandingly higher, as stated in section 3.1. It should be also noted that the increase in the current magnetic field gradient in the QMS system while using the same volume of magnet material is possible. Indeed, this optimization could be accomplished by reducing the dead volume (V_dead_) without modifying the device’s width and length. Under this scenario, the efficiency in the exploitation of the magnetic energy would be considerably higher for the QMS system. Thus, the micro-QMS exhibits an outstandingly improved performance when compared to other separator designs, which substantiates its potential for recovering magnetic catalysts at relatively high flows.

## 4 Numbering up of Micro-QMSs

The micro-QMS system designed and characterized throughout this work could find attractive applications in the pharmaceutical industry, where numerous catalyzed reactions take place. For example, the micro-QMS could be exploited by Šalić et al. in order to recover the MMC used for the oxidation of NADH in a microreactor with an oscillating magnetic field.^22,24,50^ However, the use of microfluidic platforms in industrial processes is scarce due to the huge gap between the volumetric throughputs that are required in industry compared to the ones provided by microdevices (for example, 5 μL·min^–1^ in the above-mentioned study of Šalić et al.).^24,51,52^ Increasing the volumetric throughput in microfluidic systems by enlarging their dimensions results in the fading of their key advantages, since they stem from the reduced size of microstructures. Therefore, parallelization of microchannels, which is commonly referred to as numbering up or scale out, represents a potential strategy to increase the throughput while keeping the improved features of microfluidics. Numbering up involves the parallel arrangement of numerous identical microfluidic devices in order to increase the overall throughput.^51,52^

In the pharmaceutical industry, 2, 200, and 10 000 L reactors are typically employed. The retrieval of magnetic beads downstream of a 200 L reactor involves the processing of 200 L of reaction mixture in the micro-QMS. Despite its improved performance, a single micro-QMS would require approximately 13 days in order to treat the above-mentioned volume at the maximum flow rate that provides complete particle retrieval (11 mL·min^–1^, see Figure 2b), which is industrially unaffordable. Conversely, by arranging 304 micro-QMSs in parallel, working each of them at a flow rate of 11 mL·min^–1^, a volume equal to 200 L could be processed in 1 h while entirely capturing the particles. Thereby, numbering up from 1 to 304 micro-QMSs enables the complete recovery of the beads from the desired volume during a feasible time period, thus covering the industrial demand. Further numbering up to 304 micro-QMSs would lead to a reduction of the time required for the particle retrieval stage. On the other hand, an arrangement of the conventional system could also be used to completely retrieve the magnetic beads from 200 L of reaction mixture in 1 h. To this end, the arrangement of more than 110 000 conventional systems in parallel would be required, since the maximum flow rate that can be processed in each system while achieving complete bead capture (0.5 μL·s^–1^) is considerably lower than that in the micro-QMS (183 μL·s^–1^ or 11 mL·min^–1^, see Figure 2). Despite the lower total volume of each individual conventional system (4 × 4 × 2 cm^3^ ≈ 32 cm^3^) in comparison to each QMS (5 × 5 × 5 cm^3^ ≈ 125 cm^3^), the volume of 110 000 conventional systems is 2 orders of magnitude higher than that of of 300 QMSs. This outstanding reduction in the system volume, which in turn contributes to the process intensification, substantiates the enhanced efficiency of the micro-QMS.

## 5 Conclusions

The use of MMCs has received special attention since their magnetic nature facilitates the separation and retrieval of the catalysts from the reaction media once the reaction has been completed. Such a recovery can be accomplished in microfluidic devices, thus, taking advantage of the unique features of microfluidics and covering several principles of process intensification. However, the design of efficient microfluidic-magnetophoretic systems that provide entire bead capture while processing relatively high flow rates requires the selection of the most appropriate magnet arrangement in order to maximize the magnetic force acting on the beads.

In this work, we have optimized, by using a quadrupolar magnet configuration, the retrieval of magnetic particles, which can be exploited as biocatalyst support, in a continuous-flow magnetophoretic microfluidic system (micro-QMS). More specifically, we have assessed the performance of such QMS and we have compared it with a conventional microrecovery device with similar geometrical features that operates with a single magnet. Different parameters, namely, the balance of forces acting on the particles (J), the coupling of such forces with the channel geometrical features (θ), and the exploitation of the magnetic energy for fulfilling entire recoveries (φ) have been used to characterize the micro-QMS.

According to our findings, the improved performance of the micro-QMS in comparison to the conventional microrecovery system mainly stems from the outstandingly higher magnetic field gradients that are generated in the microchannel when a quadrupolar orientation of the magnets is taken. As a result of the high magnetic gradient, 1 order of magnitude higher magnetic forces are exerted on the beads. Hence, compared to the conventional system, the complete retrieval of either (a) particles flowing at 4.5 times higher velocities or (b) 2.2 times smaller particles can be successfully fulfilled in the micro-QMS, while reducing 2 orders of magnitude the total volume of the devices when multiple QMS are arranged in parallel for treating large-scale flow rates. Particularly, recovering beads at higher velocities entails the processing of ≈360 times higher flow rates than what can be treated by the conventional system, while the capture of smaller beads involves a 55% reduction of the particle dose for providing the same surface area. Regardless of the bead size or the magnetic field gradient, the dimensionless analysis reveals that complete particle retrieval is fulfilled when the balance of the magnetic and drag forces that act on the beads (J-parameter) is 0.014, and the coupling of magnetic and fluidic forces with the geometrical features of the channel (θ-parameter) is 0.5. Additionally, we demonstrated through the φ parameter, which determines the highest fluid velocity for attaining entire particle capture, per unit of energy generated by the magnet arrangement, that the micro-QMS and the conventional system exhibit a similar efficiency in the exploitation of the magnetic energy for obtaining complete bead recovery (φ ≈ 3.4 ×10^2^ m·s^–1^·kJ^–1^). Finally, the improved performance of the micro-QMS supports its use for industrial processes, being required the use of several micro-QMS in parallel in order to satisfy a typical demand from the pharmaceutical industry. Collectively, the efficient performance of our designed micro-QMS, which meets several criteria for process intensification, has been demonstrated, thus substantiating its potential for being exploited for industrial purposes. Moreover, we have also provided the basis for further optimization of QMS systems, emphasizing the great importance of the selection of the magnet configuration for enhancing the system efficiency.

## Supporting Information Available

Figures showing the results of (1) the validation of the QMS computational model, (2) the influence of V_dead_ on both the treated flow rate and the magnetic field at the rod surface, and (3) the effect of the QMS radius on both the magnetic field gradient and the treated flow rate (PDF)
