# Supplementary material for: Recovery of Magnetic Catalysts: Advanced Design for Process Intensification
Source: Ind Eng Chem Res. 2021 Sep 22;60(46):16780–90. doi: 10.1021/acs.iecr.1c03474 (PMC8630691; doi:10.1021/acs.iecr.1c03474)
Supplement: Supplementary file 1 — ie1c03474_si_001.pdf [file ie1c03474_si_001.pdf]

Supporting Information

Recovery of Magnetic Catalysts:

Advanced Design for Process Intensification

Cristina González-Fernández[1], Jenifer Gómez-Pastora[2], Eugenio Bringas[1], Maciej Zborowski[3], Jeffrey J. Chalmers[2] and Inmaculada Ortiz[1]*

[1]Department of Chemical and Biomolecular Engineering, ETSIIT, University of Cantabria, Avda. Los Castros s/n, 39005 Santander, Spain

[2]William G. Lowrie Department of Chemical and Biomolecular Engineering, The Ohio State University, 151 W. Woodruff Avenue, Columbus, Ohio 43210, United States

[3]Department of Biomedical Engineering Cleveland Clinic 9500 Euclid Avenue, Cleveland, Ohio 44195, United States

*****Correspondence ortizi@unican.es; Tel.: +34-94-220-1585

Table of contents

Figure S1. Comparison between experimental and theoretical recoveries in a QMS system.

Figure S2. Influence of the micro-QMS dead volume (V_{dead}) on both the flow rate that can be processed and the magnetic field at the rod surface (B_{min}).

Figure S3. Effect of the QMS dimensions (r_{wall}) on the magnetic field gradient and the treated flow rate.

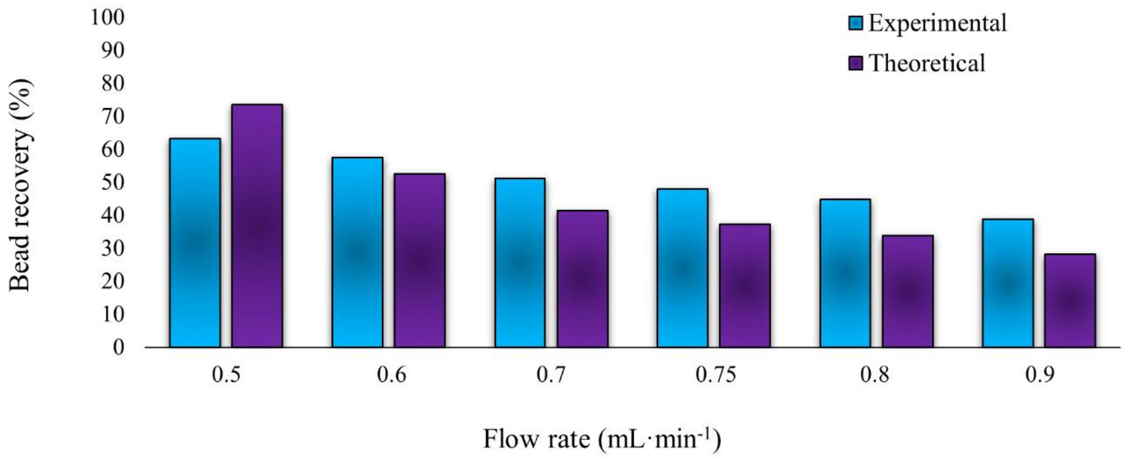

Figure S1. Comparison between experimental and theoretical recoveries in a QMS system. The QMS system employed by Moore et al. (Ref. 37 in the manuscript) for the recovery of deoxygenated red blood cells has been simulated with our numerical model. In the region of flow rates simulated, the experimental and theoretical recoveries are in good agreement. The average absolute error is less than 10% for the flow rate range evaluated and below 5% for some of the flow rate values, which confirms the validity of our numerical model.

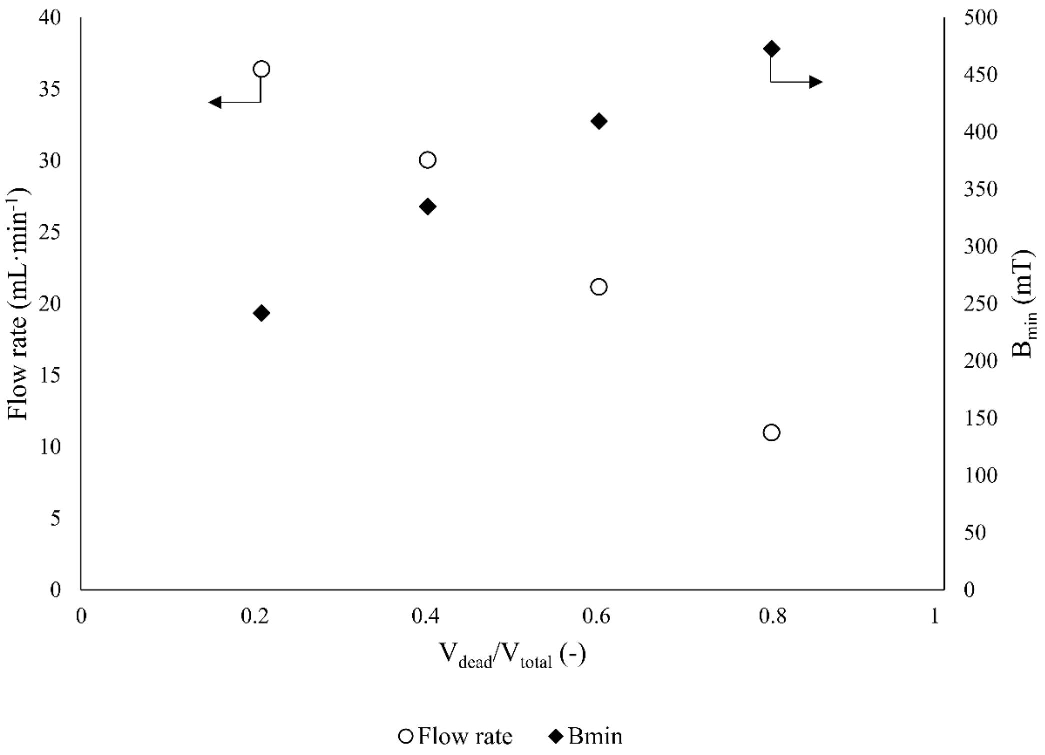

Figure S2. Influence of the micro-QMS dead volume (V_{dead}) on both the flow rate that can be processed and the magnetic field at the rod surface (B_{min}). Decreasing the r_{rod} value, and thus, the V_{dead}, increases the cross-sectional area of the device and the flow rate that can be applied. However, V_{dead}/V_{total} should be high enough to work at magnetic field values that saturate the particles (≈ 500 mT).

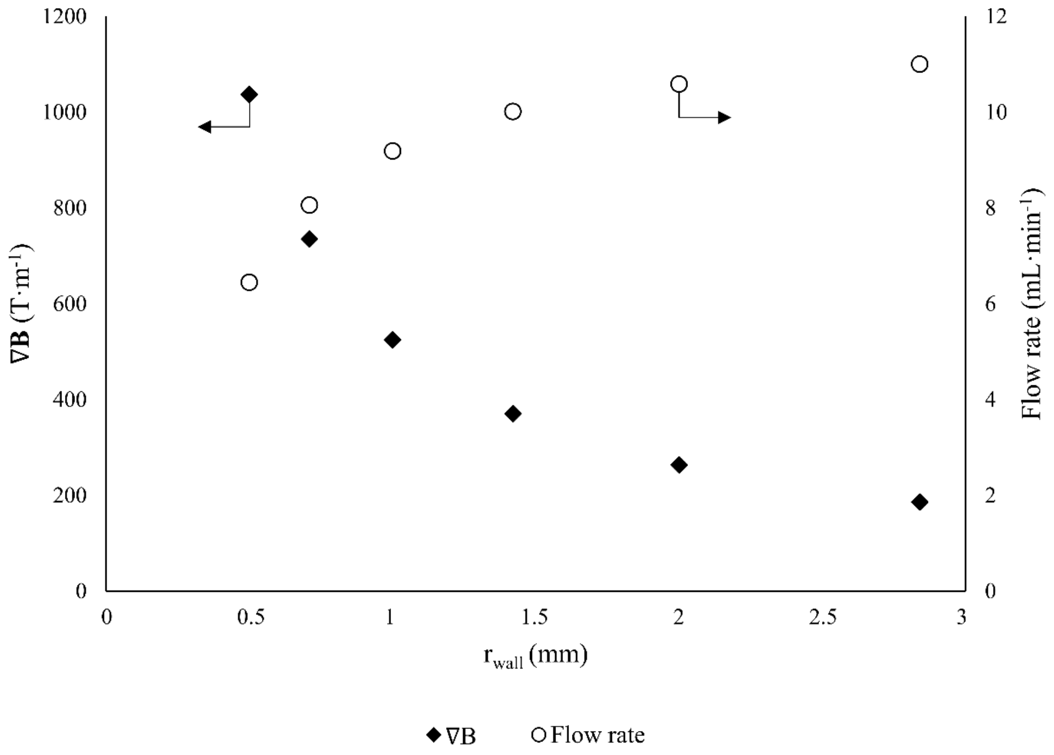

Figure S3. Effect of the QMS dimensions (r_{wall}) on the magnetic field gradient and the treated flow rate. Decreasing the r_{wall} of the QMS positively affects the magnetic field gradient achieved inside the system, however, it negatively impacts the flow rate that can be processed since the cross-sectional area depends on the r_{wall} value.
